# Arabic Mothers’ Experiences Using Complementary and Alternative Medicine for Children with Autism Spectrum Disorder: A Qualitative Study

**DOI:** 10.3390/children13010132

**Published:** 2026-01-15

**Authors:** Mais Hatahet, Attila Sárváry

**Affiliations:** 1 Doctoral School of Health Sciences, University of Debrecen, Egyetem tér 1, 4032 Debrecen, Hungary; mhatahet@hamad.qa; 2 Department of Nursing and Integrative Health Sciences, Institute of Health Sciences, Faculty of Health Sciences, University of Debrecen, Sóstói u. 2-4, 4400 Nyíregyháza, Hungary

**Keywords:** Autism Spectrum Disorder, complementary and alternative medicine (CAM), mothers, children, qualitative study, Arab countries

## Abstract

**Background/Objectives:** Autism Spectrum Disorder (ASD) is a lifelong neurodevelopmental disorder characterized by social, communication, and behavioral challenges. complementary and alternative medicine (CAM) is widely used by parents worldwide, yet research exploring parents’ experiences, particularly in Arab countries, is limited. This study explored mothers’ perceptions and experiences of CAM use for children with ASD, information-seeking behaviors and challenges encountered. **Methods**: A qualitative study using semi-structured interviews was conducted among twenty mothers at Autism Academy of Jordan in 2024. Inclusion criteria were mothers with children diagnosed with ASD for at least six months and those who had used at least one CAM therapy. Interviews were conducted via Skype, transcribed verbatim, and analyzed using NVivo 12 with inductive thematic analysis. **Results**: Three major themes emerged in this qualitative study: (1) mothers’ experiences with CAM and perceptions of benefit; (2) sources of information and decision-making processes; and (3) main challenges in selecting and implementing CAM. Mothers reported using therapies such as honey, black seed, camel milk, Hujama, olive oil, supplements, and region-specific programs like Andalosiah. Faith, cultural beliefs, and the desire for natural, safe interventions strongly influenced CAM selection. Internet searches and social media groups were primary information sources. Challenges included financial, logistical, emotional burdens, and lack of trustworthy, Arabic-language information sources. **Conclusions**: Mothers in Arab countries navigate CAM use for their children with ASD through culturally and religiously informed practices. Interventions should focus on developing evidence-based guidance, culturally sensitive counseling, and accessible information to support families in safe, informed CAM use.

## 1. Introduction

Autism Spectrum Disorder (ASD) is classified as a lifelong neurodevelopmental disorder which mainly affects behavior and communication; due to this, many therapies, practices, and methods are developing and emerging over time, which are designed to alleviate or control the associated symptoms and comorbid syndromes that are present in the majority of cases [1,2]. Parents and families of children with ASD are constantly striving to identify the most effective or comprehensive therapy options that they can offer their children to improve their quality of life [3]. The intervention process for ASD is usually going through three main paths; conventional medicine which mainly aims to control the associated behaviors and comorbid conditions, such as epilepsy, aggression, anxiety or irritability in which usually being treated by anti-psychotic medications [4,5]. Second is rehabilitation services which include occupational therapy, speech therapy, behaviors therapies, and physiotherapy. and lastly, complementary and alternative medicine (CAM) [2].

CAM refers to a broad range of health-related practices used either independently or in conjunction with conventional medical treatments, as defined by the National Center for Complementary and Integrative Health (NCCIH) [6,7]. Studies have reported that CAM is widely utilized by families of children with (ASD) worldwide, in USA 88% of large sample study have used at least one CAM during the past or recently [8]. On other studies prevalence rates ranges from 28% to 95% [9]. A recent review suggested that gut microbiota which comes under CAM, plays a role in regulating neurotransmitters, immune responses, and brain inflammation, suggesting it may be a modifiable factor influencing neurodevelopment and behavior in ASD [10]. In addition, several studies found that camel milk is a commonly used CAM intervention in ASD [11]. It contains bioactive components that may modulate antioxidant activity, and consumption of camel milk has been reported to alter antioxidant status in children with ASD correlated with improvements in behavioral outcomes, as measured by standardized assessment tools such as the Childhood Autism Rating Scale (CARS) [12,13]. Additionally, a retrospective study on hyperbaric oxygen therapy (HBOT) reported potential improvements in Verbal Behavior Milestones Assessment and Placement Program (VBMAPP) scores after six months of treatment [14]. Other studies examining dietary supplements, including vitamins, nutrients, and fatty acids, have shown reductions in associated symptoms and improvements in social and cognitive functioning [15]. However, practices like strict diet or excessive vitamin supplement found to have potential harm when parents utilize non-authorized resources, non-qualified references or service providers, unlicensed promotional products and practices, and entrusted internet information sources [16].

Parents and caregivers are facing challenges from the time they suspected something needed to be addressed, through the whole journey of therapy, and since the journey is long, many life aspects will be affected [17]. This leaves the parents in a position where they are unable to make an informed decision about whether to start the CAM therapy. They sometimes cannot decide if it is suitable or harmful, who should be consulted? Additionally, many questions that remain ambiguous may be posed without definitive responses. This all created a serious need for more scientific research based on well-defined methodologies, control groups, and longitudinal studies for better insight into the possible benefits and risks [18].

According to previous studies, parents usually tend to find solutions which are less chemical-based and more natural, products which do not have side effects as the medication, and practices which are not harmful or associated with pain [19]. A study in Europe revealed that the most mentioned CAM in literature which preferably used by parents of ASD children were dietary programs, food supplements, vitamins and minerals, music therapy, sensory integration therapy, and melatonin [20]. In Arabic region practices that were advised to be used by Prophet Mohammad or was mentioned in Qur’an and Sunnah were also prominent as a holy therapy, such as Hujama which is similar to acupuncture, the black seed, honey, camel milk, dates and others [21].

However, until recently, there has still been a gap in the topic of CAM and ASD. The research concerning CAM in Arabic countries is still in a very early phase and needs to be enhanced by further evidenced based studies [22]. Owing to the fact that addressing the actual experiences of mothers who are the main caregiver, their perceptions, beliefs and feelings cannot be fully captured by quantitative studies, this qualitative study helped to tackle in depth the perspectives and experiences concerning the use of CAM for their children with ASD [23].

## 2. Materials and Methods

### 2.1. Study Design

This study employed a qualitative research design to explore the experiences of mothers of children with ASD navigating their perspectives concerning CAM use, information sources, disclosure with doctors, and challenges in CAM use. The study used semi-structured interviews to gather in-depth data from participants, enabling rich, detailed account of their experiences.

### 2.2. Data Collection

#### Interviews and Data Collection Process

Data were collected using semi-structured, in-depth interviews with mothers of children diagnosed with ASD (Appendix A). A self-developed semi-structured interview guide was used to ensure consistency across interviews while allowing participants the flexibility to elaborate on their experiences. The interview guide was developed based on a comprehensive review of previous qualitative and quantitative studies examining CAM use among children with ASD [24] and was aligned with the aims of the present study.

The interview guide consisted of two main sections. The first section collected demographic and clinical information, including maternal age, marital status, educational level, household income, and child-related characteristics (age, age at diagnosis, specific ASD diagnosis, and comorbid conditions). The second section explored mothers’ experiences with CAM, including types of CAM used, perceived benefits and risks based on personal experience, sources of information, communication and disclosure to healthcare professionals, and mothers’ hopes, concerns, and fears regarding available therapy options (Table A1).

Interviews were conducted individually via Skype, which enabled participation regardless of geographic location and allowed mothers to engage in a familiar and private environment. Interviews were scheduled at a time most convenient for each participant to minimize burden and encourage open discussion. Each interview lasted approximately 25–30 min and was audio-recorded with participants’ permission.

Given that English was not the participants’ first language, interview questions were phrased using simple and clear language. To ensure comprehension and accuracy of responses, participants were allowed to request clarification, and questions were rephrased or translated into Arabic when necessary. This bilingual flexibility helped reduce misunderstanding and enhanced the credibility of the collected qualitative data.

### 2.3. Participant Recruitment and Selection

Participants were recruited through the Autism Academy of Jordan, and Jordanian Autism Association where mothers of children with ASD were informed about the study through direct communication by academy staff. Mothers were provided with a clear explanation of the study objectives, procedures, voluntary nature of participation, and confidentiality measures prior to enrollment.

Eligibility criteria included being the primary caregiver (mother) of a child formally diagnosed with ASD and having experience with CAM use for their child which was defined and explained to mothers who expressed interest and were contacted directly by the researcher to provide further details and arrange interviews. Participation was entirely voluntary, and mothers were informed that they could withdraw from the study at any time without consequence.

### 2.4. Time Frame

Data collection was conducted over a five-month period, from June to October 2024. This time frame allowed sufficient opportunity for recruitment, scheduling interviews, and ensuring data saturation.

### 2.5. Ethical Considerations

Ethical approval for the study was obtained from the Research and Development Department of the Autism Academy of Jordan. Given the sensitive nature of autism-related research, several ethical safeguards were implemented beyond obtaining informed consent.

Prior to participation, all mothers received detailed information about the study purpose, procedures, and benefits. Written informed consent was obtained from each participant, confirming that the data would be used solely for scientific research purposes. Confidentiality and anonymity were strictly maintained by assigning codes to participants and removing any identifying information from transcripts. Audio recordings and transcripts were securely stored and accessible only to the research team.

Participants were informed of their right to decline answering any question or to withdraw from the study at any point without any negative consequences. Emotional sensitivity was maintained throughout the interviews, and participants were given adequate time and support to express their experiences comfortably.

### 2.6. Data Analysis

Qualitative data analysis was conducted using NVivo 12 software following a thematic analysis approach [25]. All interviews were transcribed verbatim and reviewed multiple times to ensure accuracy and familiarity with the data. Initial familiarization involved repeated reading of transcripts to gain an overall understanding of participants’ narratives.

The interview guide was developed after a structured review of relevant literature investigating CAM use in ASD populations, which informed the formulation of interview questions in alignment with the study’s aims. An inductive coding approach was employed, allowing themes to emerge directly from the data rather than being imposed on a priori.

Two independent researchers conducted the qualitative analysis. Each researcher initially coded the transcripts separately, generating preliminary codes, themes, and subthemes. The researchers then met to compare findings, discuss discrepancies, and refine the thematic structure. Through iterative discussion and consensus, final themes and subthemes were agreed. NVivo’s node function was used to organize and categorize data into themes, while query and word-frequency tools supported the identification of patterns and relationships across interviews. Codes and themes were continuously reviewed and refined throughout the analysis process to ensure coherence and depth.

To enhance rigor and objectivity, reflexivity was explicitly considered. The researchers acknowledged their professional backgrounds in working with children with ASD and their families and actively reflected on how these experiences could influence interpretation. Regular peer discussions and collaborative analysis were used to minimize bias and strengthen analytical credibility.

## 3. Results

### 3.1. Sociodemographic Characteristics of the Sample

A total of twenty mothers of children diagnosed with ASD participated in the study. Their interviews were analyzed using NVivo 12. Mothers’ ages ranged from 31 to 56 years, with a mean age of 41.6 years (SD = 6.73). Most participants were married (n = 19), and most had attained tertiary education (n = 13). Household income levels varied across the sample, with most families reporting an average income, while a smaller proportion reported low- or high-income levels (Table 1).

The children with ASD ranged in age from 3 to 18 years (mean = 11.1 years, SD = 5.05), with a mean age at diagnosis of 2.4 years (SD = 0.80). Mothers described their children’s conditions using terms such as “on the spectrum,” “moderate,” or “severe autism,” reflecting both clinical understanding and subjective interpretation of symptom severity. Several mothers reported comorbid conditions, including gastrointestinal disturbances, sleep disorders, sensory sensitivities, and epilepsy, which frequently influenced their decisions to seek CAM therapies.

### 3.2. Patterns of CAM Use and Disclosure to Healthcare Professionals

All participating mothers reported using at least one form of CAM for their child with ASD. CAM practices ranged from commonly used home-based remedies (e.g., dates, olive oil, honey, black seed, food supplements, and vitamins) to less frequently used or specialized approaches, such as camel milk, donkey milk, reishi mushroom, oxygen therapy, cupping (Hujama), royal jelly, jaw deep manipulation, Sabkha application, and the Andalosiah program—a regional holistic approach emphasizing sensory connection with nature, physical affection, and reduced exposure to electronic devices (Table 2).

CAM use was deeply embedded in cultural, religious, and familial contexts, particularly for remedies perceived as “natural” or mentioned in religious texts. Dates and olive oil were universally used by all mothers, while honey and dietary supplements were also highly prevalent.

Despite widespread CAM use, most mothers (86%, n = 18) reported not disclosing CAM use to their child’s healthcare providers. Instead, mothers primarily relied on internet searches, social media platforms, and peer networks as information sources. NVivo word-frequency analysis identified terms such as “Google,” “Facebook,” “group,” “natural,” “improve,” and “hope” as among the most frequently occurring words, underscoring the central role of online communities in shaping CAM-related decision-making.

The financial cost of CAM varied substantially. While some practices were inexpensive and readily available within households, others required significant financial investment, including travel, imported products, or long-term use of supplements.

### 3.3. Thematic Findings from Qualitative Analysis

Thematic analysis yielded three overarching themes, each comprising multiple subthemes that captured mothers’ lived experiences, motivations, and challenges related to CAM use:

1. Mothers’ Lived Experiences with CAM—Perceptions of Benefit and Meaning
2. Navigating Information and Decision-Making: Sources of CAM Knowledge
3. Challenges and Barriers in CAM Use

#### 3.3.1. Theme 1: Mothers’ Lived Experiences with CAM—Perceptions of Benefit and Meaning

This theme reflects how mothers evaluated CAM therapies not only in terms of observable child outcomes but also through emotional, spiritual, and symbolic meanings. NVivo analysis revealed frequent references to “natural,” “safe,” “trust,” and “benefit.”

##### Subtheme 1.1: CAM Believed as Safe and Natural Therapeutic Option Based on Religious Tradition

Many mothers described CAM as gentle, natural, and spiritually reassuring, often contrasting these approaches with pharmacological treatments perceived as “chemical” or potentially harmful. Remedies rooted in religious tradition were particularly valued, as they provided a sense of moral and spiritual safety.

“Anything from nature can’t hurt him. Honey and black seeds are from the Prophet’s medicine, so I feel safe using them.”

(Mother 7)

Some mothers reported perceived improvements in sleep, behavior, or gastrointestinal symptoms, even when these changes were described as modest. Importantly, even minimal improvements were experienced as meaningful, reinforcing hope and emotional resilience.

“When I gave him camel milk, I saw small changes—he started sleeping better. Maybe it’s not a miracle, but it gave me hope.”

(Mother 11)

Several mothers acknowledged the complexity of attributing outcomes directly to CAM use, noting that factors such as child age, severity of ASD, comorbidities, duration of use, and concurrent therapies could influence outcomes. One mother described systematically documenting her child’s response to camel milk, illustrating parents’ attempts to independently evaluate effectiveness.

##### Subtheme 1.2: Internal Motivation to Do More for Their Children

Although many mothers of ASD children believed in natural products, uncertainty regarding the effectivity of these therapies was also evident. Some mothers continued CAM use despite inconsistent or unclear outcomes, driven by a sense of obligation to “keep trying.” This theme thus reflects a tension between faith-driven optimism and experiential doubt, with hope serving as a powerful motivator for continued CAM use.

“Sometimes I don’t see change, but I cannot stop. I feel I must keep trying until I see any improvement.”

(Mother 3)

#### 3.3.2. Theme 2: Navigating Information and Decision-Making: Sources of CAM

This theme highlights mothers’ active but largely unsupported information-seeking processes. Most mothers relied on social media platforms, online forums, and peer recommendations, often due to limited access to reliable, evidence-based Arabic-language resources.

“I learned most things from other mothers online—Facebook groups and WhatsApp.”

(Mother 10)

Although the internet was perceived as a valuable resource, it was also described as a confusing and overwhelming information mining possibility.

“The internet has everything, but everyone says something different. I’m afraid to try something not proven.”

(Mother 4)

##### Subtheme 2.1: Seeking Symptom-Specific Solutions

ASD is accompanied by a number of physical and mental symptoms. Mothers frequently sought CAM to address specific challenges such as sleep disturbances, hyperactivity, feeding difficulties, or gastrointestinal symptoms.

“I just want my child to sleep well so both of us can function. I tried anything natural that might calm him.”

(Mother 6)

Many participants expressed a strong desire for trusted, centralized sources of CAM information, emphasizing the need for guidance that integrates parental experiences with professional oversight. Despite this need, communication with healthcare professionals was limited, because the participants felt that the doctors did not support the use of CAM methods. The NVivo matrix coding revealed a negative association between CAM use and disclosure to physicians.

“Doctors don’t support these methods. They say it’s not proven, so I keep it to myself.”

(Mother 9)

Mothers with higher education levels tended to cross-check multiple sources, while others relied heavily on anecdotal advice from relatives or peers. Participants emphasized that healthcare professionals’ lack of CAM knowledge contributed to parents’ reliance on informal networks.

#### 3.3.3. Theme 3: Challenges and Barriers in CAM Use

This theme captured the main challenges and barriers (e.g., financial, emotional, cultural, and ethical burdens) associated with CAM use. The mothers who want to use one or more forms of CAM for their ASD children have to face several challenges and barriers. NVivo sentiment analysis indicated that this theme contained the highest concentration of negative emotional expressions, including fear, guilt, and exhaustion.

##### Subtheme 3.1: Financial and Logistical Burden

Some CAM therapies imposed significant financial strain, particularly when involving travel or long-term dietary interventions.

“We spent so much on diets, supplements, and travel. It’s draining.”

(Mother 5)

Families with limited financial resources tended to rely on home-based remedies, whereas higher-income families were more likely to pursue specialized or imported therapies.

##### Subtheme 3.2: Emotional Strain, Pressure, and Uncertainty

Mothers described feeling overwhelmed by competing advice from family members, cultural expectations, and social pressures. Some mothers also reported confusion and emotional distress immediately following diagnosis, which intensified their vulnerability to unverified CAM claims.

“They forced me to try a therapy for my son without consulting a doctor. I didn’t feel I had the right to say no.”

(Mother 14)

##### Subtheme 3.3: Fear of Harm and Responsibility

While mothers actively sought CAM to alleviate symptoms, many expressed fears of causing harm or interfering with conventional treatments. This fear reinforced mothers’ desire for professional guidance and underscored the ethical responsibility they felt toward their children and other families.

“If something works for my child, I can’t recommend it to others. Every child reacts differently, and I’m afraid to cause harm.”

(Mother 8)

## 4. Discussion

This study investigated the perceptions and experiences of mothers of children with ASD in Jordan who utilize CAM. Three key themes emerged from qualitative thematic analysis: mothers’ lived experiences with CAM and perceived benefits, sources of knowledge and decision-making processes, and challenges encountered during CAM selection and implementation. Collectively, these findings highlight the emotional complexity, sociocultural context, and informational gaps that influence parental decision-making. However, it is important to interpret these findings cautiously, as perceived benefits do not necessarily equate to clinically validated outcomes.

The mothers’ narratives reflected an authentic hopefulness and faith-driven motivation for their involvement with CAM. Many participants reported using therapies based on religious and cultural beliefs, such as honey, black seed, olive oil, and Hujama—practices authorized by the Qur’an and Sunnah. This finding is consistent with Masri et al. (2019) [24], who found that CAM practices in Arabic regions are frequently linked to religious identity and confidence in divine healing. Mothers considered “natural” or “non-chemical” interventions as safer alternatives to standard pharmacological treatments, matching global findings that parents of children with ASD usually chose interventions that are considered as natural and free of adverse effects [26]. Despite their optimism, mothers voiced perplexity, worry, and self-doubt, especially over whether they were making the correct decisions or endangering their child. This emotional dualism emphasizes the moral and psychological burden that mothers bear as primary decision makers and carers.

The study further revealed that mothers predominantly relied on informal information sources, particularly internet searches and social media platforms. In the era of artificial intelligence and rapid information dissemination, online communities—especially parent-led Facebook groups—were perceived as accessible and emotionally supportive [27]. This finding aligns with Alwhaibi et al. (2016), who reported that limited professional input often pushes families toward peer-driven networks [28]. Critically, while such platforms may foster shared experiences and emotional reassurance, they also increase exposure to misinformation, pseudoscientific claims, and unverified interventions. Variations in health literacy and educational background appeared to influence how parents evaluated and applied CAM-related information, which raises concerns regarding unequal risk exposure and potential harm [29].

Socioeconomic factors also emerged as influential in shaping CAM selection. Consistent with existing literature, families with lower income tended to favor traditional or community-based CAM practices perceived as affordable and accessible, whereas parents with higher educational attainment were more likely to pursue structured and commercially available CAM modalities, including dietary supplements and mind–body interventions. Within Arab societies, cupping remains deeply embedded culturally and socially, a pattern widely documented in regional studies [30,31]. However, this cultural normalization may reduce critical scrutiny of safety and appropriateness, particularly when such practices are applied to children with neurodevelopmental conditions.

Healthcare professionals are generally restricted from recommending CAM therapies unless supported by scientific evidence; nevertheless, some practitioners may offer informal advice based on clinical judgment [32]. This inconsistency can further blur boundaries between evidence-based care and alternative practices. The study highlights a pressing need for structured national awareness initiatives, clear clinical guidelines for CAM use in ASD, and safety-monitoring frameworks. Integrating evidence-based CAM education into national ASD programs could empower families with accurate information while reducing reliance on anecdotal sources [33].

Although no direct causal relationship was established, mothers’ educational backgrounds appeared to influence how they accessed, interpreted, and trusted CAM information. Difficulty understanding medical terminology often led to greater reliance on anecdotal advice from relatives or community members. This observation aligns with prior evidence linking parental education and health literacy to CAM utilization patterns [34]. Critically, this finding underscores the need for culturally sensitive, linguistically accessible educational materials that translate scientific evidence into practical guidance.

Financial, logistical, and emotional burdens were consistently reported. While some CAM modalities—such as honey, camel milk, and supplements—were affordable and widely accessible, others (e.g., oxygen therapy, specialized diets) required significant financial investment and travel. Importantly, many mothers reported limited or unclear benefits despite substantial expenditure of time and resources. This reinforces concerns raised by Khalifa et al. (2023) regarding the lack of robust, controlled trials assessing the efficacy and safety of CAM interventions in ASD [35] and highlights the ethical implications of promoting costly therapies without adequate evidence.

When compared with international literature, CAM utilization patterns in this study demonstrated both convergence and divergence. The high prevalence of CAM use parallels findings from the Middle East, Asia, and Western countries, where biologically based therapies and dietary modifications are commonly reported [36,37,38,39]. However, the prominence of culturally specific practices such as cupping reflects regional influences and underscores the importance of contextualized research rather than universal assumptions about CAM use.

Finally, beyond financial barriers, the most critical gap identified was the absence of accessible, reliable, and authoritative CAM information sources. Mothers consistently expressed the need for reputable platforms—such as verified websites, applications, or databases—offering evidence-based guidance, expert consultation, and shared experiences. This gap reflects broader deficiencies in health communication and represents an opportunity for interdisciplinary collaboration among clinicians, researchers, and parent advocacy groups [40]. Future research should adopt more comprehensive methodologies, including larger and more diverse samples to better elucidate CAM utilization patterns and support informed, evidence-based decision-making for families of children with ASD.

### Limitations

This study has some limitations that should be acknowledged when interpreting the findings. First, although the sample size was adequate for exploratory analysis, the number of participants was relatively small and regionally concentrated, which restricts the generalizability of the results to broader national or international populations. Second, all interviews were conducted in English, rather than in the caregivers’ native Arabic language. This may have inadvertently reduced the richness or nuance of responses, as participants may express thoughts and emotions more accurately in their native language. Future research should therefore incorporate Arabic interviews or bilingual data collection to minimize linguistic bias.

In addition, information was based on mothers’ self-report, which introduces potential recall bias, social desirability bias, and subjective interpretation of perceived outcomes or benefits of CAM. The sampling method also relied on voluntary participation and referrals selected only by one therapist, resulting in a sample that does not fully represent families of diverse socioeconomic or educational backgrounds, or those who do not actively seek CAM interventions. In our sample the secondary and tertiary educated mothers participated, therefore our study represents their perspectives. A more inclusive sampling framework, incorporating randomization and broader recruitment settings, would strengthen future research.

Despite these constraints, the current study offers meaningful and context-specific insights into parental motivations for CAM use, contributing valuable preliminary evidence to an area where empirical data remains limited. The findings may guide future work to adopt larger, more diverse samples and mixed-method designs to deepen understanding and support evidence-informed pediatric care.

## 5. Conclusions

Mothers with ASD children tried to use several types of CAM therapies as a proactive natural form of complementary therapy. The main difficulties applying the CAM therapies were the uncertainty of effectiveness, fear of harmful effects and the confusing information received mainly through social media. Mothers either do not disclose CAM use to their doctors, or the doctors are reluctant to discuss the use of CAM therapies with the mothers. The findings underscore that healthcare workers should prepare to engage in talks about CAM with cultural sensitivity, openness, and nonjudgmental communication, fostering safe practices while respecting families’ views. They should receive knowledge about CAM to engage in dialogs and provide evidence-based information and counsel for parents with ASD children. Developing culturally relevant training programs, guidelines and online tools for healthcare professionals and parents may assist making informed decisions while safeguarding families from exploitation or harm.

Furthermore, future research should concentrate on longitudinal and comparative studies that evaluate both the psychosocial and clinical results of CAM in ASD. Collaboration between universities, ASD centers, and policy makers would be crucial in the development of ethical, evidence-based frameworks and guidelines for CAM integration.

## Figures and Tables

**Table 1:** Sociodemographic characteristics of the sample (N = 20).

Variable	Level	N or Average (SD)
Age of mother		41.6 years (6.73)
Marital status	Married Divorced	19 1
Education of mother	Primary education level	0
	Secondary education level	7
	Tertiary education level	13
Family income level	High	4
	Average	12
	Low	4
Age of children		11.1 years (5.05)
Age at first diagnosis of ASD		2.4 years (0.80)
Comorbidity of children	Yes	4
	No	16

ASD—Autism Spectrum Disorders.

**Table 2:** Complementary and alternative methods used by mothers for children with ASD (N = 20).

CAM Practice	N (%)
Dates	20 (100)
Olive oils	20 (100)
Honey	19 (95)
Food Supplements	18 (90)
Black seeds	16 (80)
Vitamins	12 (60)
Hujama (cupping)	15 (75)
Oxygen therapy	6 (30)
Andalosiah program	5 (25)
Reishi mushroom	4 (20)
Other	5 (25)

## Data Availability

The original contributions presented in this study are included in the article. Further inquiries can be directed to the corresponding author.

## Abbreviations

The following abbreviations are used in this manuscript:

CAM	Complementary and Alternative Medicine
ASD	Autism Spectrum Disorders

## Appendix A

**Table A1:** Interview questions.

Section	Question	Details
Information About You	1. Age (if possible)?	
	2. Marital status?	
	3. Education?	
	4. Family income level?	Average, High, Low
Information About the Child	1. How old is your child?	
	2. Age at first diagnosis?	
	3. Specific diagnosis?	
	4. Does the child suffer from any disorders other than autism?	
Alternative Non-Drug Therapies	1. Have you ever tried any type of non-drug therapies? Please list them!	Examples: camel milk, oxygen therapy, music therapy, nutritional supplements, cupping, honey, any simple non-drug therapy
	2. Which of these therapies did you find to have tangible benefits?	- How did you notice the benefits? Was it in the child’s behavior, language development, or any other aspect?
		- How long did the therapy last? And was the child taking other treatments at the same time?
	3. Which of these therapies did you find to have no benefits?	
	4. Which of these therapies had adverse and non-beneficial effects?	
Sources of Information and Selection	1. What or who was your reference for choosing a non-drug therapy? Or your source of information?	
	2. Did you inform or consult your child’s doctor or therapy specialists before trying any non-drug therapy?	If not, why?
Cost of Therapies	1. In your opinion, were these therapies expensive?	Are they accessible to everyone, or do they require a lot of money, time, etc.?
Alternative and Complementary Medicine	1. If you had the option, would you prefer to avoid drug therapies and move towards alternative and complementary medicine?	If yes, what are the reasons?
Challenges	1. What were the biggest challenges you faced during your child’s treatment and rehabilitation journey?	
